# Usage of a Web-Based eHealth Intervention for Women With Stress Urinary Incontinence: Mixed Methods Study

**DOI:** 10.2196/38255

**Published:** 2022-11-17

**Authors:** Lotte Firet, Theodora Alberta Maria Teunissen, Rudolf Bertijn Kool, Kim Josephina Bernadette Notten, Antoinette Leonarda Maria Lagro-Janssen, Huub van der Vaart, Willem Jan Jozef Assendelft

**Affiliations:** 1 Department of Primary and Community Care Radboud Institute for Health Sciences Radboud University Medical Center Nijmegen Netherlands; 2 IQ Healthcare, Radboud Institute for Health Sciences, Radboud University Medical Center Nijmegen Netherlands; 3 Department of Obstetrics and Gynecology, Radboud University Medical Center Nijmegen Netherlands; 4 Department of Gynecology, University Medical Center Utrecht Utrecht Netherlands

**Keywords:** eHealth, urinary incontinence, women, usage, nonattrition, adherence, implementation science, pelvic floor muscle training, mixed methods design

## Abstract

**Background:**

Stress urinary incontinence (SUI) is highly prevalent among women and has an impact on physical and mental well-being. eHealth with pelvic floor muscle training (PFMT) has shown to be effective in reducing complaints. The usage and nonusage attrition of eHealth for SUI is unknown, but knowledge about users and their usage patterns is crucial for implementation purposes.

**Objective:**

This study aimed to evaluate how an eHealth intervention for SUI was used and by whom, explore reasons for nonusage attrition, and determine what factors are associated with usage.

**Methods:**

In this observational, mixed methods study, women with SUI independently registered to a web-based eHealth intervention, *Baas over je blaas*, a translation of the Swedish internet program *Tät-treatment of stress urinary incontinence*. Log-in data were collected during 3-month access to the website, and surveys were sent at baseline. Participants were divided into three user groups (low, intermediate, and high) and were compared based on sociodemographic and incontinence-related characteristics. Nominal logistic regression analysis was used to study factors associated with eHealth usage. Qualitative content analysis was used for open-ended questions about nonusage attrition and about facilitators of and barriers to eHealth usage.

**Results:**

Participants (n=561) had a mean age of 50.3 (SD 12.1) years, and most of them (340/553, 61.5%) had never visited a health care professional for SUI before. Most users were low users (295/515, 57.3%), followed by intermediate users (133/515, 25.8%) and high users (87/515, 16.9%). User groups differed significantly in age (48.3, SD 12 years; 52.1, SD 11.6 years; and 55.3, SD 10.9 years; *P*<.001) and in their expected ability to train the pelvic floor muscles (7.5, SD 1.4; 7.7, SD 1.4; and 8.1, SD 1.5 for low, intermediate, and high users, respectively; *P*=.006). Nonusage attrition was mainly caused by problems in integrating PFMT into everyday life. High age (>50 years), previous PFMT, and high expected ability to train the pelvic floor muscles are associated with high usage. Facilitators for eHealth usage were the clear explanation of exercises and the possibility of self-management. Barriers were its noncommittal character and the absence of personal contact.

**Conclusions:**

eHealth fulfills a need for women with SUI who have never received treatment. Those who discontinued prematurely did so mainly because it was difficult to integrate the training schedule into their everyday lives. High eHealth usage was more likely for women aged >50 years, with previous PFMT, and with high expectations about their ability to train the pelvic floor muscles. Knowledge of these user characteristics can guide clinicians and correct their misunderstandings about the suitable target population for this intervention. Furthermore, strategies for reinforcing expectations and self-efficacy are important to upscale eHealth usage, together with paying attention to people’s need for personal contact.

**International Registered Report Identifier (IRRID):**

RR2-10.2196/13164

## Introduction

### Background

Urinary incontinence is a common condition with a serious impact on quality of life and well-being [1,2]. Stress urinary incontinence (SUI) is a prevalent subtype, which is defined as the complaint of any involuntary urinary leakage on effort, exertion, sneezing, or coughing [1]. SUI affects 1 in 4 middle-aged adult women [3,4]. It can lead to psychological problems, such as fear of producing malodors that can be detected by others, shame, or even depression [2,5,6]. Furthermore, it hampers physical mobility by interfering with daily activities, causing the affected individuals to avoid work duties, sport and exercise, or social activities as these may provoke urinary leakage [6].

Pelvic floor muscle training (PFMT) is an evidence-based treatment option for SUI and is recommended as first-line treatment by a general practitioner (GP), nurse, or physiotherapist [7,8]. However, only a minority of women with SUI receive treatment because GPs tend to underdiagnose urinary incontinence and women are not likely to consult a health care professional with this problem [9]. These women feel ashamed, do not prioritize this problem, or believe that no suitable therapy is available [10-12]. Digital treatment options for SUI are promising and upcoming because they gain a broad reach by lowering the threshold to seek help [8]. Various randomized controlled trials (RCTs) and a cohort study showed that web-based (eHealth) and app-based (mobile health) self-management interventions with PFMT are effective in reducing or stopping incontinence [13-17]. Women are highly satisfied with these interventions as they give them the opportunity to deal with the problem themselves and thus promote independence [18-20].

eHealth as a self-management intervention requires users to engage with it for the recommended training period and it requires them to guide themselves throughout the program. Our previous study showed that women need to possess self-efficacy to adopt eHealth for SUI [21]. However, these requirements can be challenging, which is reflected by the high rate of nonusage in eHealth interventions [22-24]. Eysenbach [24] uses the term *nonusage attrition* to refer to the phenomenon of people prematurely discontinuing eHealth usage. Various factors could contribute to this, such as demographic factors, absence of personal or face-to-face contact, push factors (such as reminders), or external events [24]. A systematic review showed that predictors for adherence to web-based psychological interventions are being female, having high expectations, and having therapist support (eg, email support) [25].

### Objectives

Extended information on real-life usage and nonusage attrition is lacking in the case of eHealth for urinary incontinence. Knowledge of how eHealth is actually used and when and why people stop using it could lead to further improvement of the design of the intervention, and such improvements could increase usage on a large scale and thus contribute to wide implementation. Furthermore, knowledge of factors associated with usage can guide clinicians in understanding the target audience for whom eHealth would be a suitable treatment option. As part of an implementation project, this study had a three-fold aim: (1) to evaluate how an eHealth intervention for SUI is used and by whom, (2) to explore reasons for nonusage attrition, and (3) to determine what factors are associated with usage. As adherence to regular PFMT is hard to maintain, we hypothesized that, as with other eHealth interventions, nonusage attrition rates may be high [26].

## Methods

### Design

We used a mixed methods design to study the usage of an eHealth intervention for women with SUI. The quantitative strand consisted of technical log-in statistics and data from web-based questionnaires. The qualitative strand consisted of data from open-ended web-based survey questions. A detailed description of the study has been published previously [27]. The study was registered in the Dutch Trial Registry (NTR6956) prospectively, which is now included in the International Clinical Trial Registry Platform. The CONSORT-eHealth (Consolidated Standards of Reporting Trials of Electronic and Mobile Health Applications and Online Telehealth) criteria that are applicable to this study will be reported [28].

### Participants

Dutch women were recruited through news items in local newspapers, in magazines, on websites, or on social media channels between July 2018 and March 2019. Google AdWords (Google LLC) was used to make our website more retrievable. GPs in the University’s network were asked to place leaflets or posters in their waiting rooms. Women who were interested could register on the web-based eHealth intervention, and after providing informed consent, they received a short questionnaire that enabled the researcher to check their eligibility. Eligibility criteria are published in detail elsewhere [27], but, in short, women were included when aged >18 years and when having SUI or mixed urinary incontinence, which is a combination of SUI and urgency urinary incontinence [27], meaning involuntary leakage accompanied by or immediately preceded by urgency [1]. Diagnosis was based on self-assessment questions from the Questionnaire for Female Urinary Incontinence Diagnosis [29]. A woman was considered to have SUI if she replied positive to the following question: “Do you lose urine during quick moments such as coughing, sneezing, jumping, or lifting something up?” The researcher checked the eligibility criteria and then sent the web-based baseline questionnaire, which provided access to the website after completion. To use the eHealth intervention or to participate in this study, participants neither had to pay nor were they reimbursed.

### Intervention

The eHealth intervention, *Baas over je blaas*, is a translation of the Swedish eHealth intervention, *Tät-treatment of stress urinary incontinence* [30], the effectiveness of which was shown in an RCT [16,31]. It is a web-based password-protected intervention addressing PFMT. The developers of the intervention are assembled in the eContinence group from Umeå University, Sweden. They translated the program into Dutch and gave their permission to use it for research purposes through a noncommercial license agreement. The copyright of the program, Tät-treatment of stress urinary incontinence, belongs to the eContinence group at Umeå University, and the trademark is registered by the Swedish Patent and Registration office for eContinence AB, a Swedish eHealth company founded in July 2021 with the aim of maintaining, distributing, commercializing, and further developing the programs created within the research project [32]. The website was secured via HTTP Secure and hosted on the Apache web server that belonged to the Radboud University Medical Center. Data were saved in a MySQL database on the password-protected webserver. Before the study, the test version of the intervention was pilot-tested with women who varied in age, education level, and profession. Technical issues were resolved, and a new video with explanations about the program was recorded and uploaded on the log-in page. After the test phase, version 1.0 remained *frozen* during the entire study period.

**Figure 1:**
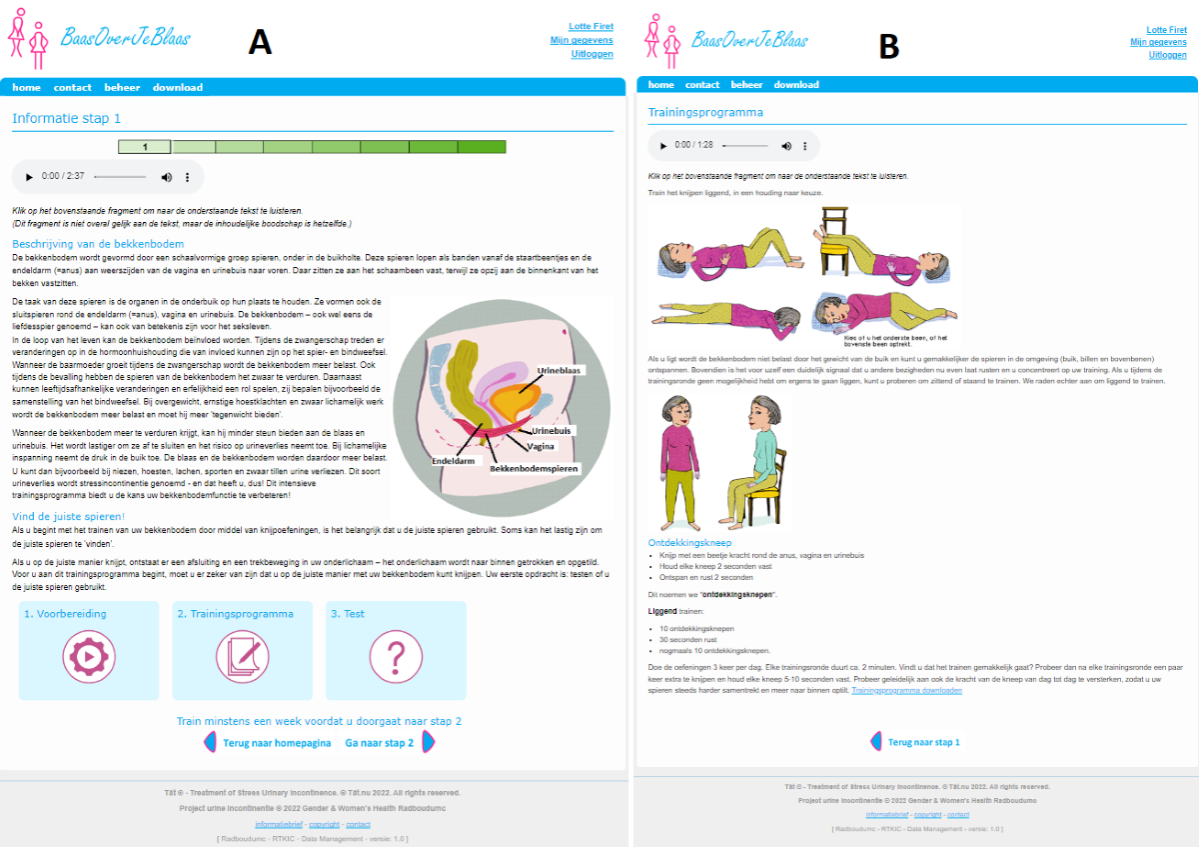
Screenshot of module number 1 (A), screenshot of training program 1 (B).

**Figure 2:**
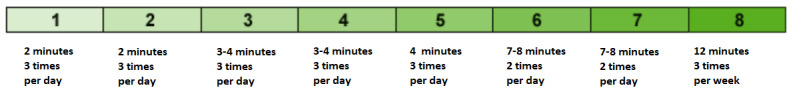
Training schedule per module.

The core content of the intervention consisted of 4 different pelvic floor muscle exercises that were addressed in 8 escalating modules with increasing intensity and complexity. The modules contained background information about incontinence and pelvic floor muscles, a training program, and a test exercise (Figure 1). Cognitive behavioral assignments were included to induce lifestyle changes. Information was provided via text, illustrations, and audio fragments and could be downloaded as PDF file. Women were advised to perform training for at least one week per module (Figure 2), and after that, the test exercise enabled them to check whether they had gained the skills required for continuation. Access to the next module was gained after the woman had completed a training report at the end of each module, which contained 2 questions about the frequency and time they spent on that module. Women were advised to consult their GP in case of no progression or if they were unable to perform the exercises.

The intervention was accessible for 3 months, and women could do the training at their own pace. A total of 3 months after the first log-in, women had 2 weeks to download the exercises of all 8 modules, and thereafter, access was closed. This restriction of access to the eHealth intervention was chosen to have a clear cutoff point for the collection of log-in data.

There was no face-to-face contact during the entire study, but the researcher was available for both content-related and technology-related questions through email (asynchronous communication). To stimulate usage, email reminders were sent if participants did not log in for 1 week, with a maximum of 2 reminders per module. Women could unsubscribe for reminders via email.

### Outcomes—Quantitative

#### Demographic and Incontinence-Related Variables

The baseline survey contained sociodemographic items (age, education level, and recruitment method) and incontinence-related items (type of incontinence, burden, duration, incontinence aid usage, previous contact with a health care professional, previous PFMT, expected ability to train the pelvic floor muscles, and expected training result). This questionnaire has been extensively described in the protocol [27]. Education level was divided into two levels: low (primary and lower secondary education) versus high (from upper secondary level to doctoral equivalent level). The expected ability to train the pelvic floor muscles was assessed on a 10-point scale ranging from 1 (very low expectations) to 10 (very high expectations) [31]. Severity of incontinence was assessed by the International Consultation on Incontinence Questionnaire–Urinary Incontinence Short Form (ICIQ-UI SF), which, based on their total score (0-21), allows women to be divided into severity categories: slight (1-5), moderate (6-12), severe (13-18), or very severe (19-21). Quality of life was assessed using the disease-specific International Consultation on Incontinence Questionnaire for Lower Urinary Tract Symptoms–Quality of Life (ICIQ LUTS-QoL), resulting in a score ranging between 19 and 76, with high score implying great impact on quality of life.

#### Usage

Log data were collected during the 3 months when participants had access to the eHealth intervention, of which the following three user parameters were defined: module number, frequency, and duration. Module number was the module that a participant had reached when access to the website was closed. Frequency was the total number of log-ins. Duration was the total number of days between the first log-in and the date on which the last training report was completed. We chose the date of the last training report instead of the last log-in date because it is a better reflection of the period in which women actively used the intervention. The last log-in date could possibly reflect women who had stopped using the intervention and logged in shortly before access to the intervention closed, to download the training program, for example. The date of the last training report could not be used for women who dropped out in module 1 because they did not complete any training report; therefore, duration for this group was the number of days between first log-in and last log-in. We corrected for false prolonged duration by choosing the previous last log-in date when women in module 1 had a duration of >90 days (12/515, 2.3%).

#### User Groups

**Figure 3:**
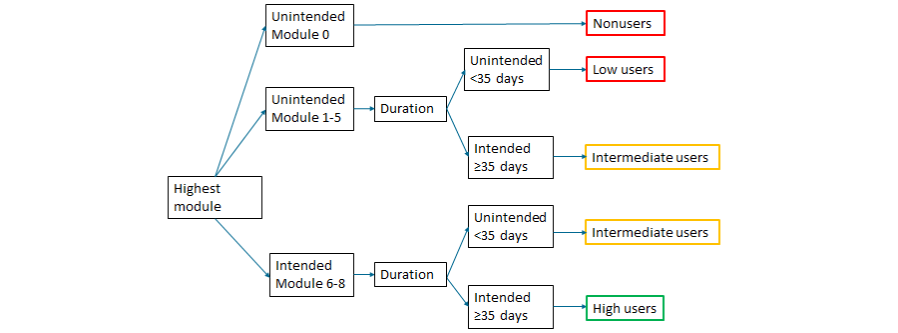
Flowchart of user groups based on module number and duration.

For the construction of user groups, the concept of *intended use* was applied, which is defined as “the extent to which individuals should experience the content (of the intervention) to derive maximum benefit from the intervention, as defined or implied by its creators” [33]. In this study, *intended use* was defined for two usage parameters: module number and duration. A module number was defined as *intended* if women completed at least module 5 because all exercises would have been addressed after 5 modules. Duration was defined as *intended* if it comprised at least 35 days, which is the multiplication of 5 modules with the recommended training duration of at least one week. On the basis of these usage parameters, we divided participants into the following user groups: non, low, intermediate, and high users (Figure 3). Nonusers completed the baseline questionnaire, but never logged in and, therefore, received no further questionnaires. Low users reached an unintended module number and had an unintended duration. Intermediate users had a combination of unintended module number with intended duration or vice versa. High users reached an intended module number and had an intended duration.

#### Adherence

Exercise adherence was defined as the percentage of time spent on PFMT out of expected time spent on PFMT and was measured using the training reports. Adherence was categorized into three levels: high (>80%), moderate (20%-80%), and low (<20%) adherence [34]. The expected time spent on PFMT was based on the prespecified training schedule (Figure 2).

### Outcomes—Qualitative: Facilitators of and Barriers to eHealth Usage and Reasons of Nonusage Attrition

After the intervention, which was 3 months after the first log-in, all women who ever logged in to the website (515/515, 100%) received a web-based survey. Nonresponders were approached via email first and then via telephone to try to collect data of all user groups. This survey contained 2 open-ended questions about facilitators of and barriers to eHealth usage (“What did you like/dislike on the program?”). These factors were studied to explore if there were additional factors associated with eHealth usage. Nonusage attrition (reasons to stop) was asked by another open-ended question to a subgroup of women who responded that they had dropped out during the intervention. The exact phrasing of this question was the following: “What was the reason for stopping prematurely OR never starting with ‘Baas over je blaas’?”

### Data Analysis

#### Quantitative Data

Data were analyzed using SPSS (version 25; IBM Corp). Descriptive statistics were calculated for usage parameters and characteristics of all user groups. As module number, frequency, and duration were not normally distributed, median and percentiles were calculated. Continuous variables were assessed using a 2-tailed, independent sample *t* test for 2 groups (nonusers vs users) and a 1-way ANOVA for 3 groups (user groups). A Pearson chi-square test was used for categorical variables. Statistical significance was determined at *P*<.05 (2-sided). Nominal logistic regression analysis was used to study factors associated with eHealth usage. Univariate analyses were performed, and variables with a significance level of *P*<.20 were included in the multivariate model. Variables were excluded stepwise in order of the highest *P* value until only statistically significant (*P*<.05) variables remained.

#### Qualitative Data

Qualitative results from 2 open-ended survey questions were analyzed through conventional content analysis [35]. First, open coding was applied to the open-ended responses. Overall, two researchers coded independently (LF and TT) and compared their codes. In case of disagreement, a third researcher (AL) gave her opinion. Codes were clustered into categories and were discussed by the research team. Data saturation was reached for all open-ended questions. Quotes are used to illustrate the findings, and they are followed by identifier number, age, and module number. The words “most,” “many,” “several,” and “a few” indicate that >50%, 20% to 50%, 10% to 20%, or <10% of respondents, respectively, shared an opinion. Microsoft Excel 2016 was the most convenient software to code the data because data were exported from SPSS.

### Ethics Approval

Ethics approval was granted by the research ethics committee of the Radboud University Medical Center, Nijmegen, the Netherlands (file number 2016-2721). The committee declared that the risks for participation in this study were negligible. The study was conducted in accordance with rules applicable in the Netherlands and the Medical Research Involving Human Subjects Act.

## Results

### Overview

**Figure 4:**
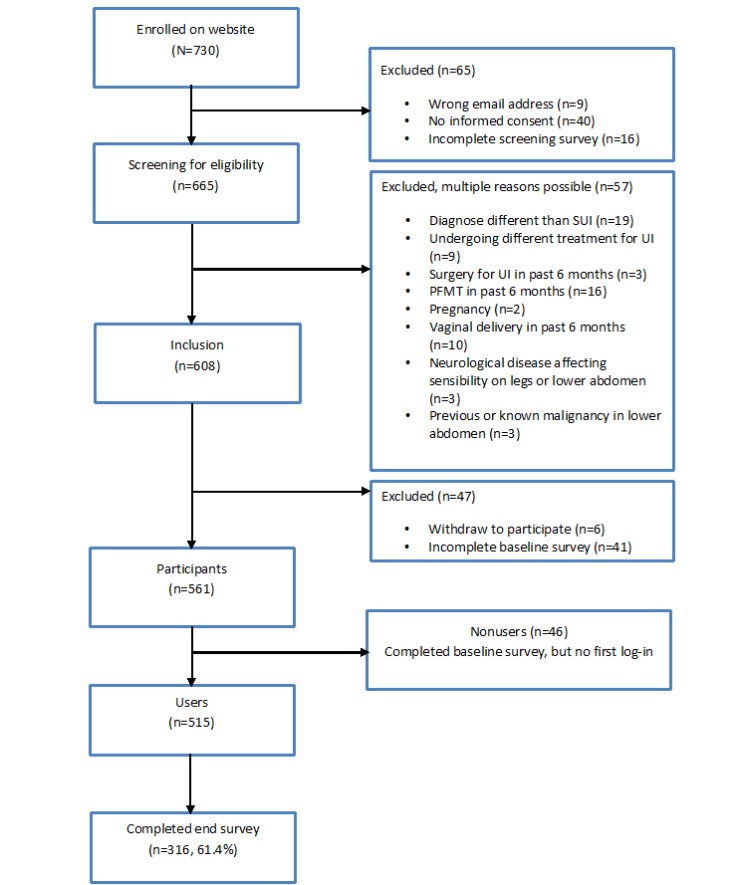
Flowchart of users and nonusers of eHealth intervention. PFMT: pelvic floor muscle training; SUI: stress urinary incontinence; UI: urinary incontinence.

A total of 730 women enrolled on the website, 608 (83.3%) of whom were included (Figure 4). Overall, 7.8% (57/730) of the women were excluded for the following main reasons: diagnosis other than SUI (mostly urgency urinary incontinence), following regular PFMT in the past 6 months, or vaginal delivery in the past 6 months. Participants (561/608, 92.3%) were women who ever logged in to the eHealth intervention, 8.2% (46/561) of whom were nonusers. Participants reached the website mostly via news items on websites or Google (244/561, 43.5%); newspapers, magazines, or newsletters (170/561, 30.3%); or social media (66/561, 11.8%).

### Baseline Characteristics

Participants (n=561) had a mean age of 50.3 (SD 12.1) years, and a minority of them had a low educational attainment level and a moderate to severe degree of incontinence (Table 1). Overall, two-thirds of them (340/553, 61.5%) had never visited a health care professional for their incontinence, and (399/557, 71.6%) had never taken previous PFMT. Most participants expected major improvement of incontinence (402/560, 71.8%) or even cure (62/560, 11.1%). There were no significant differences in demographic and incontinence-related characteristics between users (515/561, 91.8%) and nonusers (46/561, 8.2%). The burden of incontinence, duration, and incontinence aid usage between these groups (data not shown) did not differ.

**Table 1:** Characteristics of all participants (n=561).

Characteristics					Values
**Demographics**					
	Age (years), mean (SD)			50.3 (12.1)	
	**Educational attainment level,** **n (%)**				
		Low	46 (8.2)		
		High	515 (91.8)		
**Related to incontinence**					
	**Type, n (%)**				
		SUI^a^	459 (81.8)		
		MUI^b^	102 (18.2)		
	**Severity (ICIQ-UI SF^c^), n (%)**				
		Slight	42 (7.5)		
		Moderate	391 (69.7)		
		Severe	128 (22.8)		
		Very severe	0 (0)		
	Quality of life (ICIQ LUTS-QoL^d^), mean (SD)			32 (6.9)	
	Previous contact with health care professional, n (%)^e^			213 (38.5)	
	Previous PFMT^f^ for incontinence, n (%)^g^			158 (28.4)	
	Expected ability to train pelvic floor muscles, mean (SD)			7.61 (1.5)	
	**Expected treatment results, n (%)^h^**				
		Slight improvement	96 (17.1)		
		Major improvement	402 (71.8)		
		Cure	62 (11.1)		

^a^SUI: stress urinary incontinence.

^b^MUI: mixed urinary incontinence.

^c^ICIQ-UI SF: International Consultation on Incontinence Questionnaire–Urinary Incontinence Short Form.

^d^ICIQ LUTS-QoL: ICIQ for Lower Urinary Tract Symptoms–Quality of Life.

^e^Missing values were removed (8/561, 1.4%); sample size, n=553.

^f^PFMT: pelvic floor muscle training.

^g^Missing values were removed (4/561, 0.7%); sample size, n=557.

^h^Missing values were removed (1/561, 0.2%); sample size, n=560.

### Usage

Most participants (220/561, 39.2%) dropped out in module 1, and (87/561, 15.5%) reached the intended module number ≥6. The median of the log-in frequency was 4 (range 2-9), and the median of duration was 26 days (range 4-62 days).

### Adherence

**Figure 5:**
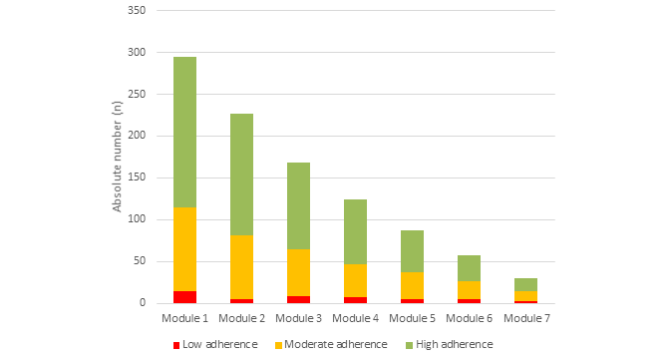
Adherence to pelvic floor muscle training, as reported in the training reports for each module.

For all modules, approximately 60% of participants had a high exercise adherence rate, as shown in the training reports (Figure 5).

### User Groups

On the basis of the intended module number and intended duration, users were divided into 3 user groups, most of whom were low users (295/515, 57.3%), followed by intermediate users (133/515, 25.8%) and high users (87/515, 16.9%). User groups differed significantly in age: the mean age for low, intermediate, and high users was 48.3 (SD 12) years, 52.1 (SD 11.6) years, and 55.3 (SD 10.9) years, respectively; *P*<.001. User groups also differed significantly in previous PFMT, with rates of 29.1% (85/292), 21.2% (28/132), and 37% (32/87) for low, intermediate, and high users, respectively; *P*=.04. The expected ability to train the pelvic floor muscles also differed significantly between user groups, with an increase in expectations from low to high users (mean 7.5, SD 1.4; mean 7.7, SD 1.4; mean 8.1, SD 1.5 for low, intermediate, and high users, respectively; *P*=.006; Table 2).

**Table 2:** Comparison between user groups in demographics and incontinence-related variables (n=515).

Variables				Low users (n=295, 57.3%)		Intermediate users (n=133, 25.8%)		High users (n=87, 16.9%)		Comparison between user groups (*P* value)	
**Demographics**											
	Age (years), mean (SD)		48.3 (12)		52.1 (11.6)		55.3 (10.9)		<.001		
	**Educational attainment level, n (%)**										.95
		Low	27 (9.2)		11 (8.3)		8 (9.2)				
		High	268 (90.8)		122 (91.7)		79 (90.8)				
**Related to incontinence**											
	**Severity (ICIQ-UI SF^a^), n (%)**										.36
		Slight	22 (7.5)		12 (9)		5 (5.7)				
		Moderate	196 (66.4)		96 (72.2)		65 (74.7)				
		Severe	77 (26.1)		25 (18.8)		17 (19.5)				
	Quality of life (ICIQ LUTS-QoL^b^), mean (SD)		32.3 (7.1)		31.5 (7)		31.4 (5.8)		.38		
	Previous PFMT for incontinence, n (%)		85 (29.1)^c^		28 (21.2)^d^		32 (36.8)		.04		
	Expected ability to train pelvic floor muscles, mean (SD)		7.5 (1.4)		7.7 (1.4)		8.1 (1.5)		.006		
	**Expected treatment results, n (%)**										.09
		Slight improvement	52 (17.7)^e^		24 (18)		9 (10.3)				
		Major improvement	218 (74.1)^e^		93 (69.9)		63 (72.4)				
		Cure	24 (8.2)^e^		16 (12)		15 (17.2)				

^a^ICIQ-UI SF: International Consultation on Incontinence Questionnaire–Urinary Incontinence Short Form.

^b^ICIQ LUTS-QoL: ICIQ for Lower Urinary Tract Symptoms–Quality of Life.

^c^Missing values were removed (3/295, 1%); sample size, n=292.

^d^Missing values were removed (1/133, 0.8%); sample size, n=132.

^e^Missing values were removed (1/295, 0.3%); sample size, n=294.

### Reasons for Nonusage Attrition

Reasons for nonusage attrition were reported in the survey, which had a response rate of 61.4% (316/515). The response rate differed significantly per user group, with a completion rate of 44.1% (130/295), 81.9% (109/133), and 90% (78/87) for low, intermediate, and high users, respectively (*P*<.001). Overall, 68% (215/316) of the participants reported that they dropped out during the intervention.

There were five categories for terminating the intervention: *mismatch between everyday life and performing PFMT*, *motivational difficulties*, *problems with execution*, *guidance needed*, and *usage of eHealth*. The most common category was *mismatch between everyday life and performing PFMT*, which was mentioned by approximately half the respondents. A mismatch was mostly caused by being very busy, forgetting to practice, (new) comorbidities or illnesses, being a caretaker for a relative, or change in daily routine (such as holidays):

I had several other physical problems that were consuming my attention, so my impatience prevented me from focusing even more on myself.

ID141; aged 62 years; module 1

A second category was that women mentioned they had *motivational difficulties* and experienced loss of motivation or lack of self-discipline. Another reason for motivational loss was either that the training had no effect or that there was an effect and the decrease in symptoms made training less urgent:

I got good results really quickly and then it all went downhill.

ID242; aged 52 years; module 4

*Problems with execution* was a third category for nonusage attrition. Several women found the exercises hard to perform or struggled with the increasing complexity of the training:

...It was pretty difficulty to do the exercises standing up. It took a while before I got the hang of this exercise. I didn’t feel the muscle, as if it wasn’t there.

ID10308; aged 52 years; module 5

A few participants mentioned that the exercises gave them bodily discomfort, such as pain in the legs or back. A minor category was *guidance needed*, with reasons such as the absence of guidance through personal contact with a caregiver and wishes for more frequent reminders:

At first I got emails to remind me, but later they stopped coming. That also made me forget where I’d got to in the program.

ID10099; aged 38 years; module 2

The last category was *usage of eHealth*, in which a few women said that they had terminated the intervention owing to technical issues or because the intervention was not user-friendly.

### Factors Associated With Usage

On the basis of univariate analysis, the following four variables were selected for the multivariate model: age (*P*<.001), previous PFMT (*P*=.04), expected ability to train the pelvic floor muscles (*P*=.005), and expected treatment results (*P*=.03). After multivariate analysis had been performed, 3 factors were significantly associated with usage (Table 3). The likelihood ratio test showed that age (*P*<.001), previous PFMT (*P*=.03), and expected ability to train the pelvic floor muscles (*P*=.008) significantly contributed to the model. Age was lower for low users than for intermediate and high users (odds ratio [OR] 0.97, 95% CI 0.96-0.99; *P*=.002 [not shown in Table 3] and OR 0.95, 95% CI 0.93-0.97; *P*<.001, respectively). The ORs for previous PFMT were lower for intermediate users than for high users (OR 0.45, 95% CI 0.24-0.83; *P*=.01). The expected ability to train the pelvic floor muscles was lower for low users than for high users (OR 0.75, 95% CI 0.62-0.91; *P*=.003).

**Table 3:** Multivariate estimates from the multinominal logistic regression model^a^.

Groups	Low users vs high users			Intermediate users vs high users	
Variables	Coefficients (SE)	Odds ratio (95% CI)	Coefficients (SE)		Odds ratio (95% CI)
Age	−0.05 (0.01)^b^	0.95 (0.93-0.97)	−0.02 (0.01)		0.98 (0.96-1)
Previous PFMT^c^	−0.35 (0.27)	0.70 (0.42-1.19)	−0.80 (0.31)		0.45 (0.24-0.83)^d^
Expected ability to train the pelvic floor muscles	−0.29 (0.10)^e^	0.75 (0.62-0.91)	−0.20 (0.11)		0.82 (0.67-1.01)

^a^Pseudo *R*^2^ (Nagelkerke)=0.093.

^b^*P*<.001.

^c^PFMT: pelvic floor muscle training.

^d^*P*=.01.

^e^*P*=.003.

### Facilitators and Barriers

The response rate on the end survey was 61.4% (316/515), of which 84.8% (268/316) and 55.1% (174/316) reported facilitators and barriers, respectively. In total, four categories emerged, including codes from both facilitators and barriers: *training instructions*, *self-management*, *usage and content*, and *effects*.

#### Training Instructions

More than half the respondents appreciated the clear explanation of the exercises, and the stepwise setup was also highly valued. Women were able to perform the exercises and felt that the instructions guided them to find the right muscles to exercise:

Clear explanation of how to squeeze and, when you can’t do it right away, reassurance that things will improve if you keep trying. That was correct in my case.

ID10326; aged 71 years; module 8

However, a few women said that the training instructions should be explained in more detail or had problems when the complexity of the exercises increased. Several women mentioned the training frequency and training duration as barriers. These women thought that the training frequency was very high and that training would be more feasible if it was lowered from 3 times to once or twice a day. The 3-month training duration was regarded as very short, and the time lock caused pressure as women who were sufficiently motivated were unable to complete all modules.

#### Self-management

Engaging in self-management treatment through eHealth was valued because women said that it provided them the flexibility to practice in their own place and in their own time and to practice by themselves without interference from a health care professional. A few women said that they liked being able to practice via the web because it provided privacy. In contrast, half of the respondents mentioned that they found eHealth to be very noncommittal and that they missed personal contact to obtain feedback on their performance or to stay motivated:

For me personally, I need more encouragement to do the exercises, working with a therapist, for instance.

ID297; aged 49 years; module 5

#### Usage and Content

Email reminders were highly valued because they made women feel guided and supported and provided them the opportunity to ask questions via email. Other content-related facilitators were the images, videos, audio fragments, download option, and possibility to write down one’s personal goals. Barriers to eHealth usage were named by a few participants: the lack of overview of the website and the lack of overview of all exercises. Others had problems in navigating through the website, reading the website on their mobile phones, or logging in repeatedly into their computers. An app would be more accessible, according to several women. Other barriers were the presence of a lot of text or redundancy and the need for more visual support.

#### Effects

Women were encouraged to continue when they noticed a positive effect on their incontinence symptoms; their ability to contract the pelvic floor muscles; or other pelvic floor symptoms, such as prolapse or urge incontinence. They also said that they had gained knowledge, become more aware of their pelvic floor, and gained confidence. Having no improvement was reported as a barrier by a few participants:

I gained self-confidence. It’s very nice to feel that I’m partly back in control.

ID120; aged 61 years; module 8

Overall, women appreciated that such a program was available, urinary incontinence was getting attention, and the problem was normalized; however, a woman said that using a website for this problem had the effect of keeping it a taboo.

## Discussion

### Principal Findings

This study shows that eHealth for SUI was mainly used by women who had never visited a health care professional for PFMT or never performed PFMT before. Although adherence to the exercises was high for all modules, most participants (295/561, 52.6%) were low users. User groups differed in age and their expected ability to train the pelvic floor muscles. Reasons for nonusage attrition were problems with scheduling and prioritizing PFMT in everyday life and with execution of the exercises. Factors that were associated with high eHealth usage were high age, previous PFMT, and high expectation of being able to train the pelvic floor muscles. Further facilitating factors for eHealth usage were clear explanation, stepwise setup, guidance by email reminders, and self-management opportunities. In contrast, its noncommittal character, absence of personal contact, and high training frequency were hindering factors.

### Comparison With Previous Studies

This study indicates that eHealth fulfills a need for women who would not turn elsewhere to deal with this problem, as reflected by two-thirds of the participants (340/553, 61.5%) who had never contacted a health care professional before. Reasons for not seeking help for this problem are well studied and often related to shame and to not recognizing SUI as a treatable problem [10-12]. eHealth fills this gap by supplying a self-management tool that increases access to care. It is known that some women prefer eHealth because it allows them to take a first step before seeking help and that most eHealth participants have not had treatment before they start [13,21].

In accordance with eHealth for other conditions, this study reports a high nonusage attrition rate, with an initial rapid decline and a remaining group of steady users [24,36]. Some women in this study reported that they stopped using the intervention because the training has an early positive effect on their symptoms. Other reasons for nonusage attrition in this study have also been reported as barriers by other studies evaluating the usage of eHealth for urinary incontinence [18-21]. These barriers are adherence challenges and problems in integrating PFMT into everyday life. The current version of the intervention addresses this by providing suggestions on how to fit the exercises into daily life, such as setting an alarm or incorporating the exercises into daily routines. Several women said that a mobile app would be more accessible and push messages on a telephone could increase usage and adherence. Another reason for abandoning the program consisted of problems with executing and insecurity about correct training, which matches our findings suggesting that previous experience with PFMT facilitates eHealth usage. As most participants (399/557, 71.6%) did not perform PFMT before, they were unable to depend on previous experiences, which may have contributed to the high nonusage attrition figure. These women may profit from more intensive (digital) contact with a health care professional throughout the eHealth program. This contact can be achieved either through digital or physical consultations on several, time fixed moments. Finally, it could be that some women start out of curiosity and lose their interest early [17]. Future studies could investigate log-in data on the views per webpage to provide insight into attractive eHealth ingredients, which could lead to further improvements.

A remarkable finding is that high age (>50 years) is associated with high usage of eHealth for urinary incontinence. This shows that women with SUI who are aged >50 years are better candidates for using an eHealth program. This is not consistent with previous findings, in which clinicians were concerned about the suitability of eHealth for older people owing to their low access to technology [37,38]. GPs thought that eHealth was more beneficial for young women with SUI because the burdens of time-consuming jobs and childcare would prevent them from visiting a health care professional [38]. Nevertheless, a recent study confirmed our findings by showing that women who were recruited through social and conventional media, for participation in an app-based intervention for urinary incontinence, were old compared with those who were recruited through their GP [39]. This indicates that concerns by GPs are incorrect because eHealth is suitable for older women also and that attention is needed before clinicians exclude women from participation. We hypothesize that women aged >50 years have few conflicts in everyday life that prevent them from continuing treatment. Another explanation could be that *older women* prefer eHealth treatment to be delivered via a website instead of a mobile app, which was also found by others [40,41]. This eHealth intervention was not mobile friendly, which was also mentioned by several women who said that it was hard to view the website on their mobile phone. If readability on a mobile phone is improved, young women may be encouraged to continue usage.

Finally, this study underlines the importance of paying attention to people’s expectations about their ability to execute the exercises. Having high expectations is associated with high eHealth usage. Although the question about the expected ability to perform PFMT is not a validated question for assessing self-efficacy, it approximates it. Self-efficacy is defined as “people’s beliefs about their capabilities to produce effects” [42]. It is known that self-efficacy expectations and attitudes to exercise are determinants of adherence to PFMT [26]. Our previous study already showed that women need a certain degree of self-efficacy to adopt the eHealth intervention for SUI [21]. In this study, one can argue whether the assessed difference in expectations about PFMT between low and high users is clinically relevant. However, studies on eHealth for urinary incontinence confirm our finding by showing that high expectations about treatment and self-rated ability to perform PFMT are determinants for treatment success [31,43]. Therefore, it is important for women to have a sense of confidence before the start of the eHealth intervention. This can be achieved either by health care professionals or by improvements in the eHealth intervention itself. Currently, the intervention starts with plain information about the effectiveness of the intervention and about goal setting options, and encouraging phrases in email reminders are used to stimulate adherence. Improving self-efficacy can be effectively achieved by including positive suggestions about eHealth before and during participation [44]. When GPs refer to eHealth, they need to enhance the women’s confidence in performing PFMT with web-based training and emphasize their ability as part of motivational interviewing [26].

### Strengths and Limitations

A major strength of this study is that it focuses on eHealth intervention usage for urinary incontinence in a real-world setting rather than in a trial setting. It is known that adherence and nonusage attrition differ for users of open access websites versus users of websites in an RCT [36]. Participants in this study had to register and complete at least the baseline survey but did not have to follow a strict research protocol, which simulated real-life usage more accurately. Another strength is that we categorized user groups based on 2 log parameters and used the term *intended use*, both highly recommended [33,45]. Using a mixed methods design enabled us to seek explanations for findings from quantitative analyses from the qualitative data, such as reasons for nonusage attrition.

A limitation of this study is that its generalizability may be restricted owing to the low proportion of participants with low education in contrast to the general Dutch population (9% vs 29%) [46]. Previous studies on eHealth for urinary incontinence showed that most participants were highly educated [13,15,16], possibly because eHealth users are generally more literate [41]. Another limitation is that adherence to PFMT exercises was assessed using self-reported training reports, which may have affected data validity because participants had to complete them before they gained access to the next module. However, the adherence to PFMT for every module was approximately 60%, and this is consistent with adherence rates to regular PFMT, which is estimated to be 64% [26]. Finally, the log-in data could have been affected by 2 aspects. First, some women may have trained offline because it was possible to download the training. Second, for research purposes, access to the intervention was restricted to 3 months. If no download option were included or more time were provided, numbers in the intermediate or high user groups may have increased.

### Conclusions

This study shows that eHealth fulfills a need for women with SUI who have never received treatment before. Although adherence to PFMT was high for every module, most participants stopped prematurely because it was difficult for them to integrate training into their everyday lives. High usage is more likely among women aged >50 years, those who received previous PFMT, and those with high expected ability to train the pelvic floor muscles. Knowledge about these user characteristics can guide clinicians and correct possible misunderstandings about the suitable target population for this intervention. Furthermore, strategies for reinforcing expectations and self-efficacy are important to upscale eHealth usage. Paying attention to people’s need for personal contact is also important; including digital methods for communicating with a health care professional or implementing eHealth into primary care (*blended care*) can enhance personal contact.

## Abbreviations

CONSORT-eHealth: Consolidated Standards of Reporting Trials of Electronic and Mobile Health Applications and Online Telehealth
GP: general practitioner
ICIQ LUTS-QoL: International Consultation on Incontinence Questionnaire for Lower Urinary Tract Symptoms–Quality of Life
ICIQ-UI SF: International Consultation on Incontinence Questionnaire–Urinary Incontinence Short Form
OR: odds ratio
PFMT: pelvic floor muscle training
RCT: randomized controlled trial
SUI: stress urinary incontinence
